# Ipilimumab and immune-mediated adverse events: a case report of anti-CTLA4 induced ileitis

**DOI:** 10.1186/s12885-015-1074-7

**Published:** 2015-03-01

**Authors:** Olga Venditti, Delia De Lisi, Marco Caricato, Damiano Caputo, Gabriella Teresa Capolupo, Chiara Taffon, Elisa Pagliara, Sofia Battisi, Anna Maria Frezza, Andrea Onetti Muda, Giuseppe Tonini, Daniele Santini

**Affiliations:** 1 Department of Medical Oncology, Università Campus Bio-Medico di Roma, via Alvaro del Portillo 200, 00128 Rome, Italy; 2 Department of General Surgery, Università Campus Bio-Medico di Roma, Via Álvaro del Portillo, 200, 00128 Rome, Italy; 3 Department of Pathology Università, Università Campus Bio-Medico di Roma, Via Álvaro del Portillo, 200, 00128 Rome, Italy; 4 Department of Radiology Università, Campus Bio-Medico di Roma, Via Álvaro del Portillo, 200, 00128 Rome, Italy

**Keywords:** Melanoma, Ipilimumab, Immune-mediated adverse events, Ileitis

## Abstract

**Background:**

Ipilimumab is a fully human monoclonal antibody directed against cytotoxic T-lymphocyte antigen-4 , a key negative regulator of T-cell activation approved by the Food and Drug Administration as of March 2011 for the treatment of metastatic melanoma. As a result of the up-regulation of the immune system, several immune-mediated adverse effects have been reported including colitis, dermatitis, hepatitis and rarely hypophysitis. The most frequent immune-mediated adverse effects described in literature include gastrointestinal toxicity such as diarrhea, colitis and case of colitis and ileitis.

**Case presentation:**

In this paper we report an interesting case of immune-mediate ileitis without colitis in a 54 years old woman with metastatic melanoma treated with ipilimumab. We also discuss about case management and the possible pathological mechanisms considering also previous reports.

**Conclusions:**

The aim of this article is to support further investigations concerning epigenetic and genetic analysis in order to personalize biological therapy and to reduce immune related adverse events observed after ipilimumab administration.

## Background

Ipilimumab is a fully human monoclonal antibody directed against cytotoxic T-lymphocyte antigen-4 (CTLA-4), a key negative regulator of T-cell activation approved for the treatment of metastatic melanoma. The antibody contributes to activate immune response against tumor cells exploiting this mechanism of action. However, Ipilimumab treatment has been associated with severe and potentially life-threatening immunological adverse effects due to T cell activation and proliferation. Most of the serious adverse effects affect gastro-intestinal tract and include diarrhea, colitis and colitis associated with ileitis. In this paper we describe a case of immune-mediate ileitis without colitis observed in a 54 years old woman with metastatic melanoma after 3 cycles of treatment with ipilimumab. We also report histological and CT findings showing respectively extensive superficial ulceration, full-thickness inflammatory infiltrate and indirect sign of ileum wall necrosis. These findings demonstrate how an immune system disregulation induced by Ipilimumab may result in severe and fatal immune-mediated adverse reactions caused by T-cell activation and proliferation.

## Case presentation

A 54 years old woman with previous history of malignant cutaneus melanoma came to our attention in April 2013. In 2009 she had undergone surgery after the detection of a nodular mass located in the right iliac fossa. Pathology specimens were consistent with pigmented nodular melanoma, with vertical growth, Clark level III, Breslow tickeness of 9 mm. The excision margins were negative. Right inguinal sentinel lymph node was positive but a subsequent dissection excluded further lymphnodal involvement (stage IIIB: pT4a, pN1a, pMx). A staging Fluorodeoxyglucose-PET (FDG-PET) showed no local or distant disease. Then patient started a follow up program until January 2012, when a CT scan showed multiple bilateral lung metastases and a FDG-PET scan revealed pathological uptake in the right iliac fossa and lumbar vertebra L3. Mutational analysis of BRAF V600E was carried out with the detection of exon 15 mutation. The patient started a first line treatment with Dabrafenib (150 mg orally twice daily) from May 2012 to February 2013. The FDG-PET scan performed in October 2012 proved a complete metabolic response in right iliac fossa and a partial response at L3 level; moreover, the CT scan demonstrated a reduction in number and size of lung metastases. In January 2013 a new FDG-PET scan showed progressive disease in lung and bones (D11, L3, 4th right rib and pelvis) and appearance of liver metastases. Subsequently patient started a second line treatment with Ipilimumab (3 mg/kg every 3 weeks planned for a total of 4 doses). On June 2013 two days after the third cycle of Ipilimumab she was admitted to our oncology unit reporting fever (body temperature >38.5°C) without shiver, Grade 2 asthenia and nausea and Grade 1 diarrhea. The blood cultures performed were negative for bacterial growth. Administration of intravenous Metilprednisolone 2 mg/Kg die was started with clinical benefit but without a complete resolution of fever. Considering the significant clinical benefit obtained, patient was dismissed with prescription of oral prednisone 1 mg/kg daily. Given previous toxicity and according to the drug schedule, the 4th dose of ipilimumab was delayed until the toxicities returned to Grade 0 or 1. After two weeks, in July 2013 the patient was admitted again to our oncology unit for persisting and deteriorating Grade 3 asthenia, Grade 2 nausea and vomiting, Grade 3 diarrhea and dehydratation, despite of oral corticosteroid treatment.

**Figure 1:**
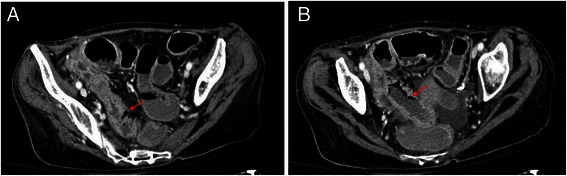
**CT axial image during venous phase.** Figure **A** shows a marked wall thickness (arrow) at the level of pre terminal ileum. Figure **B** shows a thicker wall of the ileum, with the presence of air (arrow), which is a typical sign of *pneumatosis* intestinalis, the lume is enlarged. These finding suggest the presence of necrosis of the ileum wall.

Physical examination proved a poor hydration of skin and mucosal membranes, a diffuse abdominal pain, in particular in the right iliac fossa; blood examination revealed anemia (Hemoglobin- Hgb- 9.60 g/dL) and hypoalbuminemia (1.74 g/dL). During the hospital stay fluids, corticosteroids (metilprednisolone 2 mg/Kg die) and albumin were administered with partial resolution of symptoms. Few days later, clinical conditions worsened, because of the development of gastrointestinal (GI) hemorrhage, severe anemia (Hgb 5.20 g/dL), thrombocytopenia (platelets 52.00 ×10^3/uL) and coagulation impairment (INR 1.58). Red blood cells, platelet and plasma were transfused with poor benefit. A CT scan of the abdomen was performed in order to exclude an immune-mediated colitis (described in literature as a possible side effect occurring during ipilimumab treatment [1,2]) since no comorbidities such as intestinal bowel disease or hematological disorder were referred at the moment of hospital admission. Radiological imaging showed colic mucosal hyperemia and submucosal edema (Figure 1)*.*

**Figure 2:**
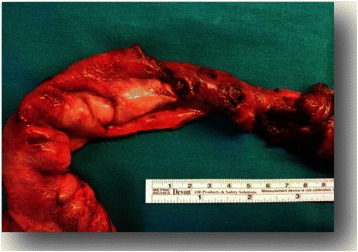
**The picture show the last tract of ileum resected; as seen in this image the small bowel appearing necrotic and perforated in several points for at least 40 cm of length.**

**Figure 3:**
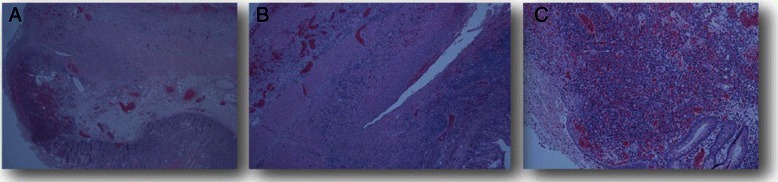
**Terminal ileum biopsies of the last small bowel tract resected.** Pathology showed extensive superficial ulceration (panel **B**) and full-thickness inflammatory infiltrate rich of lymphocytes, granulocytes and eosinophils, associated with acute serositis and vessels rupture (panel **A** and **C**).

**Figure 4:**
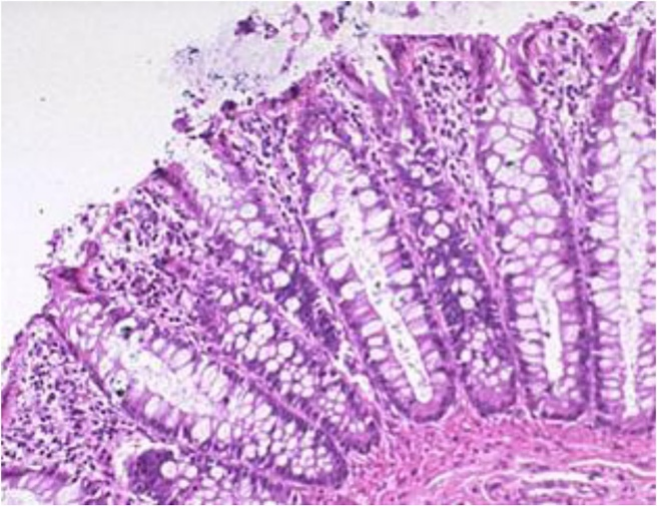
**Colon biopsy from the colonoscopy.** The pathology report describes normal colonic mucosa fragment with colic glands without inflammatory infiltrate or ulceration.

In consideration of CT findings, a total colonscopy was performed detecting red blood in rectal ampulla and sigmoid colon, without mucosal alterations and a hyperemic and edematous colic mucosa with extensive loss of substance. Terminal ileum biopsies confirmed the presence of superficial loss of substance, and an intense inflammatory infiltrate rich of lymphocytes and granulocytes with sporadic cryptic abscesses extending up to the muscolaris mucosae tonaca. Since gastrointestinal (GI) bleeding did not stop with medical therapy, the patient underwent surgery. A subtotal colectomy with resection of the last tract of terminal ileum was performed, with the small bowel appearing necrotic and perforated in several points for at least 40 cm of length (Figure 2)*.* Pathology report showed extensive superficial ulceration and full-thickness inflammatory infiltrate rich of lymphocytes, granulocytes and eosinophils, associated with acute sierosytis and vessels rupture (Figure 3). None of these findings were observed in the colic tract, where it has been found normal colic mucosa and glands without inflammatory infiltrate or ulceration (Figure 4). The patient continued steroid therapy with metilprednisolone 2 mg/Kg iv die, and no further episodes of diarrhea or GI bleeding were reporter after surgery. A post-operative CT scan showed disease progression in all sites previously detected and appearance of diffuse peritoneal carcinomatosis. In consideration of these findings, the patients was addressed to a palliative care program.

## Discussion and conclusions

Treatment of stage IV melanoma patients needs to be discussed by interdisciplinary tumor board in centers with broad experience for the disease.

Several studies investigating the mechanism of the pathogenesis identified multiple pathways to be involved in cellular invasion and drug resistance [3,4].

Cytotoxic drugs such as dacarbazine (DTIC), temozolomide, taxanes, fotemustine [5] and platin are currently considered valid choices in the management of metastatic melanoma even if new therapeutic strategies such as immunotherapy using Ipilimumab or anti-PD1 antibodies, selective BRAF inhibitors like vemurafenib and dabrafenib, c-Kit inhibitors and MAPK/ERK kinase (MEK) inhibitors have demonstrated an impressive antitumor activity in clinical trials.

Ipilimumab is a fully human monoclonal antibody directed against cytotoxic T-lymphocyte antigen-4 (CTLA-4), a key negative regulator of T-cell activation. CTLA4 is constitutively expressed on inhibitory CD25+ CD4+ regulatory T cells (Treg) and plays a key role in Treg control of the immune response. CTLA-4 thereby works as a physiologic “brake” on the activated immune system. Monoclonal antibody against CTLA-4, such as Ipilimumab, prevent this feedback inhibition, enhancing the immune response against the tumor [6]. A disregulation of the immune system induced by Ipilimumab may produce severe and fatal immune-mediated adverse reactions due to T-cell activation and proliferation. Potentially, any organ system may be involved but the most common reported irAEs are enterocolitis, hepatitis, dermatitis (including toxic epidermal necrolysis), neuropathy, and endocrinopathy.

In a phase III trial demonstrating an increase in survival, immune-related adverse events occurred in approximately 60 percent of patients treated with ipilumumab; they usually tended to appear after several weeks of treatment. Overall, severe or life-threatening (grade 3 or 4) toxicities were seen in 10 to 15 percent of ipilimumab-treated patients, compared to 3 percent in those receiving only gp100. The most frequent irAEs described in literature include GI AEs such as diarrhea and colitis.

In the same study, severe, life-threatening, or fatal (diarrhea of 7 or more stools above baseline, fever, peritoneal signs; Grade 3–5) immune-mediated enterocolitis occurred in 34 (7%) patients and moderate (diarrhea with up to 6 stools above baseline, abdominal pain, mucus or blood in stool) Grade 2 both ileitis and colitis occurred in 28 (5%) Ipilimumab-treated patients. Across all Ipilimumab-treated patients (n = 511), 5 (1%) patients developed intestinal perforation, 4 (0.8%) patients died as a result of complications, and 26 (5%) patients were hospitalized for severe enterocolitis [7].

Although clinical presentation is similar to inflammatory bowel disease (IBD), lesions distribution and pathological characteristics are different from Crohn’s disease (CD), ulcerative colitis (UC) or graft-versus host disease. The predominantly diffuse nature of the active inflammation in colonic biopsies from patients after onset of diarrhea or colitis are similar to ulcerative colitis but features of chronicity and diffuse colonic involvement distally (hallmarks of UC) were not observed. Not of all were the distinctive features of CD, including granulomas, aphthous or fissuring ulcers, and bowel wall thickening secondary to transmural inflammation. CD is primarily a disease of the proximal colon and terminal ileum whereas the majority of abnormal histologic findings in patient treated with Ipilimumab were located distally; finally, the histologic findings observed were also distinct from graft-vs.-host disease, which is characterized by prominent epithelial apoptosis and glandular destruction [8].

Berman D. et al. tried to explain these different pathological findings in a recent study showing that blockade of CTLA-4 by Ipilimumab may cause disregulation of GI mucosal immunity as pointed out by fluctuating antibody titers against enteric flora, increased levels of neutrophil-derived fecal calprotectin, and immune infiltration into the mucosa. This autoimmune deregulation is completely different from those observed for classic IBD; the pattern of positive antibody against enteric flora observed in this study was not consistent with that for classic UC or CD. In CD, approximately half of patients are positive for anti-Saccharomyces cerevisiae antibody (ASCA), CBir flagellin antibody (anti-CBir1), and anti-I2, and <25% are positive for perinuclear antineutrophil cytoplasmic antibody (pANCA); in UC, approximately half of patients are positive for pANCA, but <10% are positive for ASCA, anti-CBir1, anti-I2, and antiOmpC [9]. In this study, the most common positive titers in patients with grade 2 or higher irAEs were to pANCA and antiOmpC, with <10% of patients positive for anti-I2 and ASCA, and <15% positive for anti-CBir1.

Finally, the fluctuating antibody titers observed in this study were also inconsistent with CD, where titers are stable over time and despite changes in disease activity. This fluctuation may reflect changes in the state of T-cell activation as Ipilimumab concentrations cross an unidentified threshold. The parallel and transient changes in antibody levels in conjunction with an increase in fecal calprotectin are consistent with dysregulated mucosa induced by CTLA-4 blockade in these non-IBD patients [10].

The autoimmune disregulation reported by some patient treated with iplimumab can be explained by common genetic variation in the CTLA4 gene. Sanderson et al., hypothesized that the GG allele of rs7565213 (JO30), reported to have lower CTLA4 activity, correlates with a higher change of developing autoimmune symptoms and subsequently could be associated with improved prognosis. In their study of 19 patients, 3 out of 4 patients with the GG allele developed autoimmune symptoms [11]. Despite these interesting data, a subsequent study did not confirm this hypothesis since a significant association between the G allele of re7565213 (JO30) and the development of autoimmune reactions was not observed [12].

In the present case report we describe an interesting case of immune-mediate ileitis without colitis secondary to Ipilimumab administration.

We strongly suggest clinician to consider inflammatory ileitis in differential diagnosis when the patients present persistent diarrhea/ bloody diarrhea and abdominal pain in the right iliac fossa when colitis is ruled out with colonoscopy, in order to better optimize surgical treatment if necessary.

Since the patient developed only ileitis without colitis - as reported in the most of patient in the phase III study - we can suppose that two different autoimmune pathways could be activated against colic and ileal epitopes.

Further data are needed to establish which ileal cell epitopes and which specific subpopulation of the enteric flora was the main target of Ipilimumab damage. A better understanding of Ipilimumab immune related pathway with the view of identifying a specific immune T cell subpopulation involved in these adverse reactions would also be of great interest.

## Consent

Written informed consent was obtained from the patient for publication of this Case report and any accompanying images. A copy of the written consent is available for review by the Editor of this journal.
